# Concurrent BMP Signaling Maintenance and TGF-β Signaling Inhibition Is a Hallmark of Natural Resistance to Muscle Atrophy in the Hibernating Bear

**DOI:** 10.3390/cells10081873

**Published:** 2021-07-23

**Authors:** Laura Cussonneau, Christian Boyer, Charlotte Brun, Christiane Deval, Emmanuelle Loizon, Emmanuelle Meugnier, Elise Gueret, Emeric Dubois, Daniel Taillandier, Cécile Polge, Daniel Béchet, Guillemette Gauquelin-Koch, Alina L. Evans, Jon M. Arnemo, Jon E. Swenson, Stéphane Blanc, Chantal Simon, Etienne Lefai, Fabrice Bertile, Lydie Combaret

**Affiliations:** 1 INRAE, Unité de Nutrition Humaine, Université Clermont Auvergne, UMR 1019, F-63000 Clermont-Ferrand, France; christian.boyer@inrae.fr (C.B.); christiane.deval@inrae.fr (C.D.); daniel.taillandier@inrae.fr (D.T.); cecile.polge@inrae.fr (C.P.); daniel.bechet@inrae.fr (D.B.); etienne.lefai@inrae.fr (E.L.); 2 Université de Strasbourg, CNRS, IPHC UMR 7178, F-67000 Strasbourg, France; charlotte.brun@etu.unistra.fr (C.B.); stephane.blanc@iphc.cnrs.fr (S.B.); fbertile@unistra.fr (F.B.); 3 CarMen Laboratory, INSERM 1060, INRAE 1397, University of Lyon, F-69600 Oullins, France; emmanuelle.loizon@inserm.fr (E.L.); emmanuelle.meugnier@univ-lyon1.fr (E.M.); chantal.simon@univ-lyon1.fr (C.S.); 4 Institut de Génomique Fonctionnelle (IGF), University Montpellier, CNRS, INSERM, 34094 Montpellier, France; elise.gueret@mgx.cnrs.fr (E.G.); emeric.dubois@mgx.cnrs.fr (E.D.); 5 Montpellier GenomiX, France Génomique, 34095 Montpellier, France; 6 Centre National d’Etudes Spatiales, CNES, 75001 Paris, France; guillemette.gauquelinkoch@cnes.fr; 7 Department of Forestry and Wildlife Management, Inland Norway University of Applied Sciences, Campus Evenstad, NO-2480 Koppang, Norway; alina.evans@inn.no (A.L.E.); jon.arnemo@inn.no (J.M.A.); 8 Department of Wildlife, Fish, and Environmental Studies, Swedish University of Agricultural Sciences, SE-901 83 Umeå, Sweden; 9 Faculty of Environmental Sciences and Natural Resource Management, Norwegian University of Life Sciences, NO-1432 Ås, Norway; jon.swenson@nmbu.no

**Keywords:** brown bear hibernation, mouse unloading, muscle atrophy, physical inactivity, RNA sequencing, TGF-β/BMP signaling

## Abstract

Muscle atrophy arises from a multiplicity of physio-pathological situations and has very detrimental consequences for the whole body. Although knowledge of muscle atrophy mechanisms keeps growing, there is still no proven treatment to date. This study aimed at identifying new drivers for muscle atrophy resistance. We selected an innovative approach that compares muscle transcriptome between an original model of natural resistance to muscle atrophy, the hibernating brown bear, and a classical model of induced atrophy, the unloaded mouse. Using RNA sequencing, we identified 4415 differentially expressed genes, including 1746 up- and 2369 down-regulated genes, in bear muscles between the active versus hibernating period. We focused on the Transforming Growth Factor (TGF)-β and the Bone Morphogenetic Protein (BMP) pathways, respectively, involved in muscle mass loss and maintenance. TGF-β- and BMP-related genes were overall down- and up-regulated in the non-atrophied muscles of the hibernating bear, respectively, and the opposite occurred for the atrophied muscles of the unloaded mouse. This was further substantiated at the protein level. Our data suggest TGF-β/BMP balance is crucial for muscle mass maintenance during long-term physical inactivity in the hibernating bear. Thus, concurrent activation of the BMP pathway may potentiate TGF-β inhibiting therapies already targeted to prevent muscle atrophy.

## 1. Introduction

Muscle atrophy is defined as a loss of muscle mass and strength and is associated with adverse health outcomes, such as an autonomy decline and an increase in morbidity and mortality in many catabolic conditions (e.g., cancer cachexia, heart, and kidney failure, fasting, sepsis, injury, aging, or physical inactivity, etc.) [1,2,3,4,5]. Given the increase in sedentary behavior and improvement in life expectancy, and with to date still no proven therapeutic or preventive treatment to date, muscle atrophy remains a major public health issue (World Health Organization data) [6]. Skeletal muscle tissue represents an important reservoir of amino acids, which are mobilized during catabolic situations to preserve vital functions, resulting in an imbalance of contractile protein turnover (i.e., proteolysis exceeding protein synthesis) [7,8]. Catabolic stimuli (e.g., oxidative stress, endoplasmic reticulum disturbances, nutrient shortage, mitochondrial disruptions, etc.) activate a complex network of intracellular modulators, which in turn lead to the activation of the ubiquitin-proteasome system (UPS) and autophagy [9,10]. These two main proteolytic systems in muscle tissue involve a set of genes, i.e., atrogenes, whose expression at the mRNA levels is commonly altered during atrophy [11]. Cascades of events and players of muscle atrophy are well described and conserved in mammals [12,13] and include the transforming growth factor-β (TGF-β) superfamily, with TGF-β signaling acting as a negative regulator, and Bone Morphogenetic Proteins (BMP) signaling as a positive regulator of muscle mass [14]. The TGF-β pathway mediates muscle atrophy through cytoplasmic and nuclear signaling molecules SMAD2/3, mainly leading to the expression of the atrogenes TRIM63 (MuRF1) and FBXO32 (atrogin-1) [15]. Constitutive expression of SMAD3 triggers muscle wasting, and inhibition of SMAD2 and SMAD3 is sufficient to induce muscle growth in vivo [16,17,18,19]. Conversely, the BMP pathway mediates muscle mass maintenance through cytoplasmic and nuclear signaling molecules SMAD1/5/9, promoting a negative transcriptional regulation for a ubiquitin ligase required for muscle wasting, FBXO30 (MUSA1) [20], and increasing the expression of BMP receptors activity in muscles induced hypertrophy through Smad1/5-mediated activation of mTOR signaling [21].

Hibernating bears (Ursidae family) are naturally resistant to muscle atrophy when facing the two major atrophic inducers, prolonged fasting and physical inactivity up to 5–7 months [22,23]. Conversely and during a shorter period, a loss of muscle mass and volume prevails in rodent models [24,25,26,27,28,29] and humans [4,5]. As in rodent models, the muscles of the active bear are sensitive to disuse after denervation, whereas the muscles of the hibernating brown bear (*Ursus arctos*) are resistant [30]. The hibernating brown bear, therefore, appears as a suitable model to study the underlying mechanisms of muscle mass maintenance [31]. How it withstands muscle loss in conditions where muscle atrophy is expected in non-hibernating mammals remains to be fully elucidated. However, several hypotheses can be raised; our recent analysis of muscle proteome in the hibernating brown bear revealed the maintenance of glycolysis and a turning down of ATP turnover [32]. In addition, we reported (i) a myogenic microRNA signature prone to promoting muscle regeneration and suppressing ubiquitin ligase expression in bear muscle during winter [33], as well as (ii) limited levels of oxidative stress [34]. To unravel the molecular basis of muscle maintenance at the mRNA level, the bear muscle transcriptome has already been explored using cDNA microarrays [35] or RNA sequencing [36,37]. These two transcriptomic studies suggested an overall reduction in energy and protein metabolism, consistent with metabolic suppression and lower energy demand in skeletal muscle during hibernation. Although these studies focused on the changes in bear muscle transcriptome during the hibernating versus active period, our study aimed to compare them with those that occur in the muscle transcriptome of disuse-induced atrophy in a mouse model. The rationale for such a comparative analysis of two contrasted situations of muscle atrophy or maintenance lies in the identification of potential new candidates, beyond already reported metabolic factors [35,36,37], that may help the hibernating bear resist atrophy, thereby providing new targets for fighting muscle atrophy in humans. Among the transcription factors involved in the regulation of the differentially expressed genes highlighted in bear muscle between the hibernation and active periods, eight were involved in the regulation of the TGF-β superfamily. We therefore subsequently focused on an in-depth analysis of the TGF-β and BMP intracellular pathways.

## 2. Materials and Methods

### 2.1. Animal Experiments

#### 2.1.1. Bear Sample Collection

Biopsies from the vastus lateralis muscle were collected from 17 free-ranging brown bears, 2–3 years old (*Ursus arctos*; 11 females and 6 males), from Dalarna and Gävleborg counties, Sweden, from 2014 to 2019 (Table S6). The samples were immediately frozen on dry ice until storage at −80 °C. In a given year, the same bears were captured during winter hibernation (February) and recaptured during their active period (June). The study was approved by the Swedish Ethical Committee on Animal Experiment (applications Dnr C3/2016 and Dnr C18/2015), the Swedish Environmental Protection Agency (NV-0741-18), and the Swedish Board of Agriculture (Dnr 5.2.18–3060/17). All procedures complied with Swedish laws and regulations. Capture, anesthesia, and sampling were carried out according to an established biomedical protocol [38].

#### 2.1.2. Mouse Model of Hindlimb Unloading

Our objective was to compare the muscle transcriptome of the hibernating bear with that in a rodent model of long-term physical inactivity. We chose the 10-day mouse model of unloading as an established disuse-atrophy model, with atrophic pathways still activated [28,39]. All experiments were conducted with the approval of the regional ethics committee (agreement n° D6334515) following the European Directive 2010/63/EU on the protection of vertebrate animals used for experimental and scientific purposes. This study was performed with 12 C57BL6/J adult male mice purchased from Janvier Labs (Le Genest-Saint-Isle, France). They were housed by pairs upon arrival in a polycarbonate cage in a controlled room (22 ± 2 °C, 60% ± 5% humidity, 12 h light/dark cycle, light period starting at 8 h), fed ad libitum, and given free access to water.

After 10 days of acclimatization, the mice were either kept unsuspended (Control, *n* = 6) or subjected to hindlimb unloading through tail suspension (Unloaded, *n* = 6) for 10 days. Custom tail suspension cages were adapted from previous studies [40,41]. The cages (43 × 29 × 24 cm) have an overhead frame to which two suspension systems are fixed in parallel on the width of the cage. These suspension systems are widely spaced in a cage so that mice can always be housed in pairs without touching each other. Unloaded mice had a metal ring attached near the base of their tails using surgical adhesive tape. This ring was then attached to a swivel that allowed a 360-degree rotation and was attached on a rail that covered the upper width of the cage. The height of the swivel has been adjusted to keep the mouse at an angle of about 30° from the head so that the hindlimbs could not touch the ground or the walls. During the 10 days of unloading, mice showed only a very small body weight loss (<7%) that occurred within the first 3 days, with no change in food intake. At the end of the experiment, soleus muscles were rapidly dissected out and immediately frozen in liquid nitrogen and stored at −80 °C until analyses. As for the data used from the RNA sequencing of Zhang et al. [42], the soleus muscle atrophied by 37% (from 7.24 ± 0.27 mg in control mice to 4.58 ± 0.31 mg in unloaded mice, *p* < 0.001 according to the unpaired Student’s *t*-test).

### 2.2. RNA Sequencing of Brown Bear Muscle

#### 2.2.1. RNA Isolation

Total RNA from bear muscles was isolated as described [43]. Briefly, muscle RNA from six bears (paired samples collected in summer and winter in a given year for the same individual) was extracted using TRIzol reagent (Invitrogen, Courtaboeuf, France).

#### 2.2.2. Illumina RNA Sequencing, Data Assembly, Statistical Analysis

We constructed RNA-Seq libraries with the Truseq stranded mRNA sample preparation kit from Illumina and sequenced them in two lanes on an Illumina HiSeq2500 (single-end, 50 bp, six libraries per lane). Image analyses and base calling were performed using the HiSeq Control Software (v2.2.70, Illumina, San Diego, CA, USA) and Real-Time Analysis component (v1.18.66.4, Illumina, San Diego, CA, USA). Demultiplexing was performed using Illumina’s conversion software (bcl2fastq 2.20). The quality of the raw data were assessed using FastQC from the Babraham Institute and the Illumina software SAV (Sequencing Analysis Viewer, Illumina, San Diego, CA, USA). A splice junction mapper, TopHat 2.1.1 [44] (using Bowtie 2.3.5.1 [45], Johns Hopkins University, MD, USA), was used to align the RNA-Seq reads to the *Ursus arctos* genome (GCA_003584765.1 ASM358476v1 assembly downloaded from NCBI) with a set of gene model annotations (GCF_003584765.1_ASM358476v1_genomic.gff downloaded on 17 June 2019, from NCBI). Final read alignments having more than three mismatches were discarded. Samtools (v1.9) (http://samtools.sourceforge.net) was used to sort the alignment files. Then, the gene quantification was performed with Featurecounts 1.6.2 (http://subread.sourceforge.net/) [46]. As the data were from a strand-specific assay, the read had to be mapped to the opposite strand of the gene (-s 2 option). Before statistical analysis, genes with less than 30 reads (cumulating over all the analyzed samples) were filtered out. Differentially expressed genes were identified using the Bioconductor(https://bioconductor.org/) [47] package DESeq2 1.26.0 [48] (R version 3.6.1 https://www.r-project.org/). Data were normalized using the DESeq2(https://bioconductor.org/packages/release/bioc/html/DESeq2.html, accessed on 16 June 2021) normalization method. Genes with an adjusted *p*-value below 5% (according to the Benjamini–Hochberg procedure that controls the FDR) were declared differentially expressed.

#### 2.2.3. Functional and Pathway Enrichment Analysis

**Figure 1:**
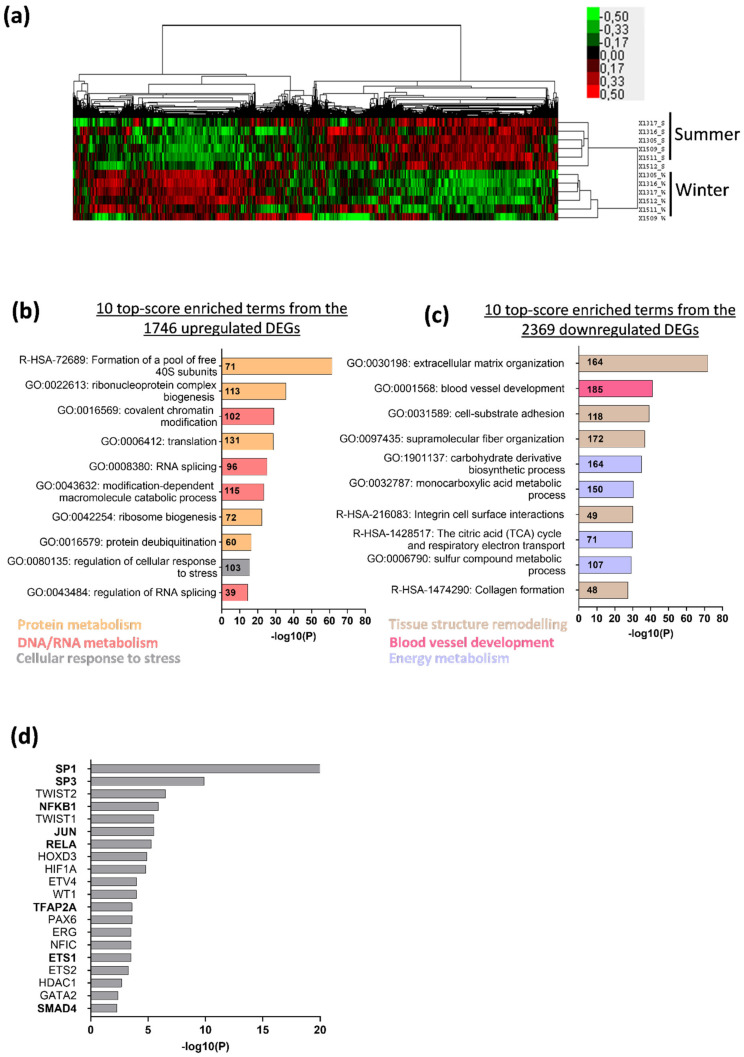
Deep changes in brown bear muscle transcriptome during hibernation. (**a**) Heatmap from brown bear muscle (vastus lateralis) transcripts (*n* = 6 bears/season, the same individuals were sampled and analyzed in summer and winter); red indicates high and green indicates low expression level of the 13531 genes. (**b**) Graph representing the 10 top-score of significantly enriched terms in winter compared to summer, from the 1746 up-regulated differentially expressed genes (DEGs) (FC W/S >|1.3| and padj < |0.01|) or (**c**) from the 2369 down-regulated DEGs (FC W/S < |0.77| and padj < |0.01|; Table S1). The color code for the functional cluster is indicated in the respective graphs, and the bold numbers in the different bars represent the numbers of DEGs found in the enriched terms. (**d**) Graph representing the 10 top-score of transcription factors involved in DEGs regulation from “Formation of a pool of free 40S subunits” and “Extracellular matrix organization” enriched terms. The bold TFs are involved in TGF-β superfamily regulation.

Hierarchical clustering of bear transcriptomic data (log-transformed) was performed using Cluster v3.0 software (University of Tokyo, Tokyo, Japan) from the 13531 reads [49]. Parameters were set as follows: median centering and normalization of genes for adjusting data and centroid linkage clustering for both genes and arrays. Dendrograms were generated and viewed using the Java Treeview v1.3.3 program (Alok Saldanha, Stanford University, Stanford, CA, USA) [50]. To identify the differentially expressed genes (DEGs), we selected a fold change (FC) Winter/Summer >|1.3| or <|0.77| and an adjusted *p*-value < |0.01| as cut-off standards, for the up- and down-regulated genes, respectively. Visualization of functional enrichment was performed using Metascape [51], a web-based portal for visualizing the inference of enriched biological pathways among the DEGs. For the given DEGs gene list, pathway and process enrichment analysis has been carried out with the following ontology sources: KEGG Pathway, GO Biological Processes, Reactome Gene Sets, Canonical Pathways, CORUM, TRRUST, DisGeNET, PaGenBase, Transcription Factor Targets, WikiPathways, PANTHER Pathway, and COVID. All genes in the genome have been used as the enrichment background. Terms with a *p*-value < 0.01, a minimum count of 3, and an enrichment factor >1.5 (the enrichment factor is the ratio between the observed counts and the counts expected by chance) are collected and grouped into clusters based on their membership similarities. More specifically, *p*-values are calculated based on the accumulative hypergeometric distribution, and q-values are calculated using the Benjamini–Hochberg procedure to account for multiple testings. Kappa scores are used as the similarity metric when performing hierachical clustering on the enriched terms, and sub-trees with a similarity of >0.3 are considered a cluster. The most statistically significant term within a cluster is chosen to represent the cluster. The 10 top-score enrichment terms from that analysis are shown in Figure 1b,c, and the 10 top-score enrichment transcription factors regulating the DEGs are shown in Figure 1d. The heat map representing the expression changes of the TGF-β/BMP gene sets in bear versus mouse muscles was made using the Pheatmap package (R 1.4.1106, University of Tartu, Tartu, Estonia). Briefly, the gene hierarchical clustering is based on Euclidean distance calculated from the log2FC value (Winter/Summer and Unloaded/Control for bear and mouse muscles, respectively).

### 2.3. Transcriptomic Data Assembly and Statistical Analysis of Mouse Muscle

We used transcriptomic data from an already published study [42]. Briefly, in this study, C57BL6/J adult male mice were either kept unsuspended (Control, *n* = 4) or subjected to hindlimb unloading through tail suspension (Unloaded, *n* = 4) for 10 days. The fastq files of eight soleus muscles were downloaded from GEO (GSE102284). A splice junction mapper, TopHat 2.1.1(Johns Hopkins University, MD, USA) [44], was used to align the RNA-Seq reads to the mouse genome (UCSC mm10) with a set of gene model annotations (genes.gtf downloaded from UCSC on 29 October 2019; GeneIDs come from the NCBI gene2refseq file). Final read alignments having more than three mismatches were discarded. Samtools (v1.9, http://www.htslib.org/) was used to sort the alignment files. Then, the gene quantification was performed with Featurecounts 2.0.0 (http://subread.sourceforge.net/) [46]. As the data were from a strand-specific assay, the read had to be mapped to the opposite strand of the gene (-s 2 option). Before statistical analysis, genes with less than 20 reads (cumulating all the analyzed samples) were filtered out. Differentially expressed genes were identified using the Bioconductor [47] package DESeq2 1.26.0 [48] as previously described (cf. 2.2.2).

### 2.4. Western Blot

Vastus lateralis muscles from eleven bears (paired samples collected in summer and winter in a given year for the same individual; Table S6) and soleus muscles from 10-days control or unloaded mice (*n* = 6/group) (~30 mg) were used. Samples were homogenized using a polytron in 1 mL of an ice-cold buffer (10 mM Tris pH 7.5, 150 mM NaCl, 1 mM EDTA, 1 mM EGTA, 1% Triton X-100, 0.5% Igepal CA630) containing inhibitors of proteases (Protease Inhibitor Cocktail) and phosphatases (1 mM Na_3_VO_3_, 10 mM NaF) (Sigma, Saint-Quentin-Fallavier, France). The homogenates were stirred for 1 h at 4 °C and then centrifuged at 10,000 *g* for 15 min at 4 °C. The resulting supernatants were then stored at −80 °C until use. The concentration of proteins was determined using the Bradford Protein Assay Kit (Biorad, Marnes-la-Coquette, France). Proteins were then diluted in Laemmli buffer and stored at −80 °C until use. Protein extracts were subjected to SDS-PAGE (sodium dodecyl sulfate-polyacrylamide gel electrophoresis) using TGX™ FastCast™ 10% Acrylamide gels (Biorad, Marnes-la-Coquette, France) and transferred onto a PVDF membrane (Hybond P, Amersham, England). Blots were blocked for 1 h at room temperature with 5% bovine serum albumin in TBS buffer with 0.1% Tween-20 (TBS-T, pH = 7.8), then washed thrice in TBS-T and incubated (overnight, stirring, 4 °C) with appropriated primary antibodies against SMAD1/5 (PA5-80036, Thermo Fisher, Illkirch, France), SMAD2/3 (#8685, Cell Signaling Technology, Saint-Cyr-L’Ecole, France), SMAD4 (ab230815), CTGF (ab227180), and GDF5 (ab137698) (Abcam, Cambridge, United Kingdom). Blots were then washed and incubated for 1 h with an appropriate secondary horseradish peroxidase-conjugated antibody at room temperature. Signals were detected after incubation with Luminata Crescendo Western HRP substrate (Millipore, Burlington, MA, USA) and visualized using G: BOX ChemiXT4 (XL1) imaging system (Syngene, Frederick, MD, USA). Signals were then quantified using the GeneTools software (Syngene, Cambridge, UK) and normalized against the total amount of proteins determined by TGX signals to correct for uneven loading. Protein data were presented as individual values. The bilateral ratio paired Student’s *t*-test was used to compare the muscles of bears during summer and winter (S and W, respectively). For muscles of control and unloaded mice (C and U, respectively), statistical significance was determined using the bilateral unpaired Student’s *t*-test. Statistical analysis was performed using Prism 8 (GraphPad Prism 9, San Diego, CA, USA).

## 3. Results

### 3.1. Deep Changes in Brown Bear Muscle Transcriptome during Hibernation

The brown bear transcriptome data set revealed that, from 13531 transcripts commonly identified in all individuals, gene expression differed markedly between summer and winter (Figure 1a). We identified 4115 differentially expressed genes (DEGs) between muscles of the active and hibernating bear with mainly down-regulated genes (Table S1). The 10 top-score obtained from an annotation enrichment analysis performed from the down- and up-regulated DEGs highlighted several significant enriched terms regulated differentially in bear muscles between the two seasons (Figure 1b,c). For instance, the protein metabolism functional cluster was the most up-regulated in bear muscles in winter compared to summer, with “Formation of a pool of free 40S subunits”, “Ribonucleoprotein complex biogenesis”, or “Translation” enriched terms (Figure 1b). In addition, the tissue structure remodeling functional cluster was the most down-regulated in muscles of the hibernating versus active bear, with “Extracellular matrix organization”, “Cell-substrate adhesion”, or “Supramolecular fiber organization” enriched terms (Figure 1c). We then run a transcriptional regulatory network analysis to identify transcription factors (TFs) involved in the DEGs regulation from the two most differentially enriched terms between the two seasons (e.g., “Formation of a pool of free 40S subunits” and “Extracellular matrix organization”) (Figure 1d). We found that SP1 was the TF the most involved in the DEGs regulation, as well as others TFs such as SP3, NFKB1, JUN, RELA, TFAP2A, ETS1, or SMAD4. Interestingly, these TFs cited above are all involved in the regulation or signal transduction of the TGF-β superfamily [52,53,54]. We therefore decided to focus on that superfamily, including (i) TGF-β signaling, which is a master regulator of the extracellular matrix organization and also involved in muscle mass loss, and (ii) BMP signaling, which has recently been discovered to be involved in muscle mass maintenance [20].

### 3.2. Hibernation Induces a Transcriptional Shift from the TGF-β to the BMP Pathway

From a thorough analysis of the literature [55,56,57], we have drawn up a list of the actors and regulators of the TGF-β superfamily. We then analyzed precisely how they were regulated at the mRNA level in the muscles of the hibernating bear between the winter and summer seasons.

**Figure 2:**
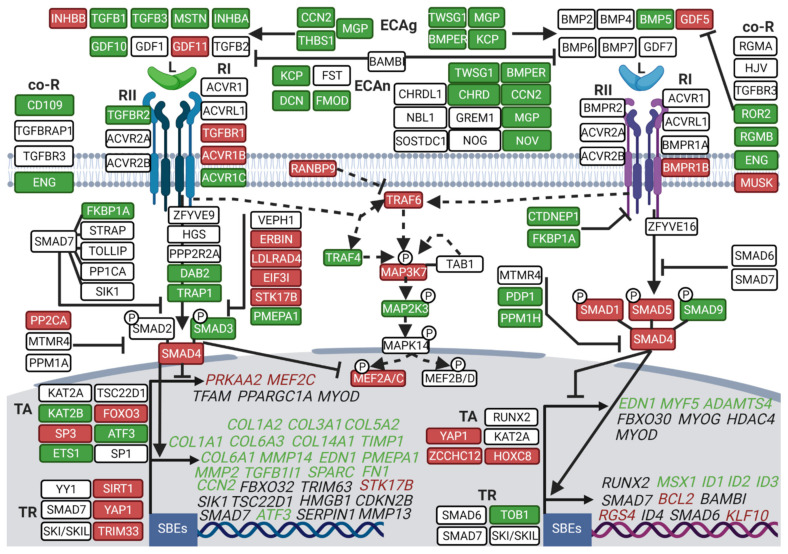
Deep transcriptomic reprogramming of TGF-β and BMP pathways in muscle during brown bear hibernation. Scheme showing brown bear vastus lateralis muscle transcripts involved in TGF-β and BMP signaling and depicting (i) their relationships [55,56,57] and (ii) the difference in their expression levels between hibernation and activity periods. Red and green boxes indicate, respectively up- and down-regulated genes during hibernation compared to the summer season, and white boxes indicate unchanged genes. Target genes of the TGF and BMP pathways are indicated in italic and are in green when down-regulated, in red when up-regulated, and in black when unchanged. Dashed lines show the SMAD-independent pathway and full lines the canonical signaling pathway. Arrows indicate activation, and ⊥ bars indicate inhibition. (*n* = 6 bears/season, the same individuals were sampled and analyzed in summer and winter, padj < |0.05|; Tables S2 and S3). ECAg: Extracellular Agonist, ECAn: Extracellular Antagonist, L: Ligand, Co-R: Co-Receptor, RII: Receptor type II, RI: Receptor type I, TA: Transcriptional Activator, TR: Transcriptional Repressor, SBEs: SMAD Binding Element. Created with BioRender.com.

The expression levels of two main TGF-β ligands, INHBA and MSTN, were dramatically lower (Fold change (FC) = 0.28 and 0.52, respectively) in winter, whereas INHBB expression was higher (FC = 1.85) (Figure 2, Tables S2 and S3). In BMP signaling, the main ligand described in muscle mass maintenance, GDF5, showed higher levels (FC = 2.5) in hibernating bear muscles compared to active muscles. Extracellular actors inhibiting (KCP, DCN, MGP, NOV, or CHRD) or promoting (CCN2 or BMPER) TGF-β and/or BMP signals were mainly down-regulated in winter compared to summer. Receptors from the TGF-β signaling were differentially expressed during hibernation, with ACVR1C and TGBBR2 levels being considerably lower (FC = 0.28 and 0.80, respectively) in winter compared to summer, whereas TGFBR1 and ACVR1B were higher (FC = 1.42 and 1.47, respectively). The GDF5 receptor, BMPR1B, was higher in winter compared to summer (FC = 1.37). The co-receptors that control intensity and specificity of the downstream TGF-β/BMP signaling were mainly down-regulated or unchanged in winter for both pathways, except for MUSK, an important BMP co-receptor in muscle cells, which was up-regulated (FC = 2.39). Overall, actors involved in the initiation of the TGF-β signal were mainly repressed while those driving the BMP signal were increased.

For TGF-β signaling, intracellular inhibitors such as ERBIN, LDLRAD4, EIF3I, STK17B, and PP2CA were up-regulated in winter bear muscles (FC = 1.26, 1.50, 1.37, 1.86, and 1.56, respectively), whereas some of the actors promoting the signal were lower expressed, i.e., DAB2 and TRAP1 (FC = 0.50 and 0.63, respectively). By contrast, for BMP signaling, intracellular inhibitors were mainly lower expressed in muscles of the hibernating bear, such as CTDNEP1, FKBP1A (FC = 0.70 and 0.67, respectively). Regarding the intracellular actors triggering TGF-β/BMP signaling, SMAD3 (TGF-β signaling; FC = 0.76) was less expressed, SMAD1 and SMAD5 (BMP signaling; FC = 1.99 and 1.30, respectively), and SMAD4 (common to TGF-β and BMP signaling; FC = 1.34) were more expressed in muscles of the hibernating bear compared to the active one. Thus, expression changes of the intracellular actors again suggest repression of TGF-β signaling but maintenance of the BMP signaling.

For nuclear components, transcriptional activators were either up- (FOXO3 and SP3, FC = 1.68 and 1.70) or down-regulated (e.g., KAT2B, ATF3 and ETS1, FC = 0.55, 0.46, and 0.54) for TGF-β, whereas mainly up-regulated in winter for BMP with YAP1, ZCCHC12, and HOXC8 (FC = 1.63, 2.21, and 2.46, respectively). Conversely, the transcriptional repressors were mainly up-regulated for the TGF-β pathway, i.e., TRIM33, YAP1, and SIRT1 (FC = 1.60, 1.63, and 1.82, respectively), whereas unchanged or lower expressed, i.e., TOB1 (FC = 0.78) for BMP during hibernation. Considering TGF-β target genes, an overall down-regulation was observed in winter compared to summer, as highlighted for the different collagen isoforms or the multiple metalloproteinases COL1A1/2 (FC = 0.04 and 0.07), COL3A1 (FC = 0.06), COL5A2 (FC = 0.28), COL6A1/3 (FC = 0.22 and 0.20), COL14A1 (FC = 0.14), and MMP2/14 (FC = 0.33 and 0.46) (Figure 2 and Table S2). For BMP target genes, it was less contrasted, with either an unchanged (e.g., RUNX2 or ID4), down-regulated (e.g., ID1 or ID2, FC = 0.31 and 0.65), or up-regulated expression (RGS4 or KLF10, FC = 1.72 and 1.32) during hibernation, with RGS4 being a muscle-specific gene. Overall, this supports a general down-regulation of the transcriptional activity that drives TGF-β signaling, with possible maintenance of that for BMP signaling.

Finally, TGF-β and BMP signaling also use a shared SMAD-independent pathway, involving a branch of the MAPK (Mitogen-Activated Protein Kinase) pathway [58]. In this pathway, the expression of TRAF6 and its downstream actor MAP3K7 were higher in muscles of the hibernating bear compared to the active one (FC = 1.78 and 1.60, respectively). Some of the TRAF6 downstream actors, including MEF2A and MEF2C, which are key muscle transcription factors, were as well up-regulated (FC = 1.89 and 2.23) in winter compared to summer bear muscles (Figure 2).

**Figure 3:**
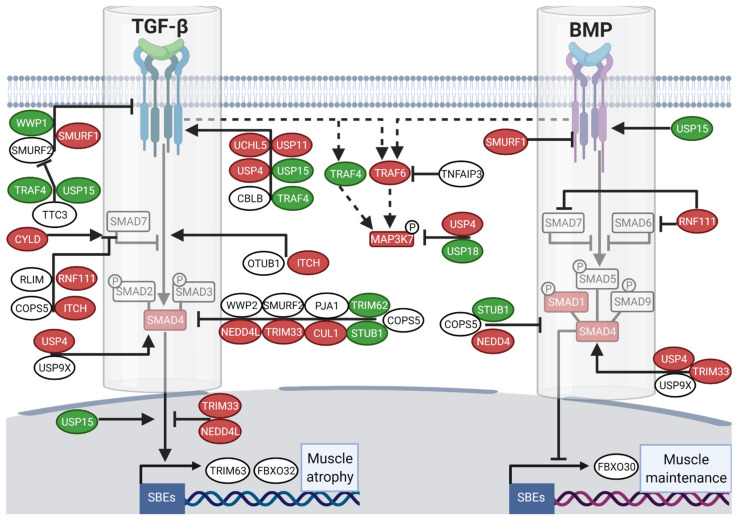
A transcriptomic reprogramming of UPS components involved in TGF-β and BMP regulation prevails in muscle during brown bear hibernation. Scheme showing the E3s/DUBs enzymes regulation of brown bear vastus lateralis muscle transcripts involved in TGF-β and BMP signaling pathways and depicting (i) their relationships [12,60] and (ii) the difference in their expression levels between hibernation and activity periods. Red and green boxes indicate, respectively up- down-regulated genes during hibernation compared to the summer season, and white boxes indicate unchanged genes. Dashed lines show the SMAD-independent pathway and full lines the canonical signaling. Arrows indicate activation and bars inhibition. (*n* = 6 bears/season, the same individuals were sampled and analyzed in summer and winter, padj < |0.05|; Table S4). SBEs: SMAD Binding Elements. Created with BioRender.com.

TGF-β and BMP signaling pathways are tightly regulated by several UPS members, such as E3 ubiquitin ligases (E3s) and deubiquitinating enzymes (DUB) [55,59]. TGF-β inhibitors localized from the receptors to the nucleus level were up-regulated in winter, such as for CUL1 (FC = 1.85), NEDD4L (FC = 1.63), and TRIM33 (FC = 1.60), the latter being also a positive regulator of BMP signal transduction (Figure 3 and Table S4). SMURF1, an E3 ligase inhibiting both TGF-β and BMP signaling, was also up-regulated (FC = 1.64) in muscles of the hibernating bear. For UPS activators of the TGF-β signaling pathway alone, several DUBs were up-regulated in winter, e.g., UCHL5, USP11, and USP4 (FC = 1.78, 1.77, and 1.37, respectively), the latter also promoting BMP signaling, and others were down-regulated such as for USP15 and TRAF4 (FC = 0.52 and 0.47). The TGF-β and BMP pathways regulate the transcription of some muscle-specific E3s (TRIM63, FBXO32, and FBXO30). None of them were differentially expressed in winter compared to summer (Figure 3 and Table S4).

Overall, this wide transcriptomic analysis highlighted a winter transcriptional pattern in muscles of the brown bear that was prone to shutting down TGF-β signaling while maintaining or even over-activating the BMP pathway.

### 3.3. Divergent Regulation of TGF-β and BMP Pathways in Atrophy-Resistant Muscles of the Hibernating Brown Bear versus Atrophied Muscles of the Unloaded Mouse

**Figure 4:**
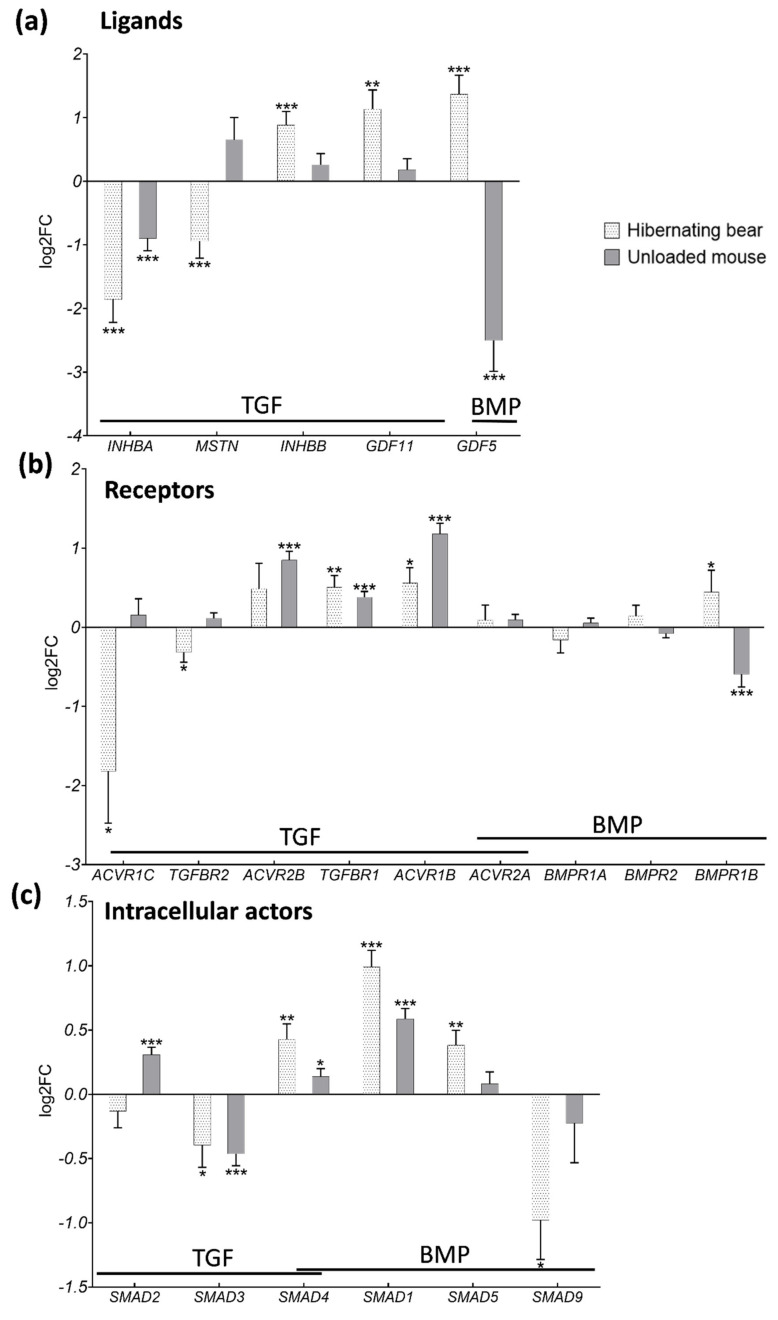
The gene expression pattern of TGF-β and BMP components is different in brown bear muscle resistant to atrophy during hibernation compared to atrophied muscles of the unloaded mouse. Genes expression level in vastus lateralis muscle of active and hibernating brown bears (*n* = 6 bears/season, the same individuals were sampled and analyzed in summer and winter, log2FC Winter/Summer, dotted white bars) and in soleus muscle of control and unloaded mice (*n* = 4 mice per condition, log2FC Unloaded/Control, gray bars). The depicted genes were categorized as TGF-β and BMP (**a**) ligands, (**b**) receptors, and (**c**) intracellular actors. Data are expressed as log2FC ± lfcSE. Statistical significance is shown (* padj < |0.05|; ** padj < |0.01|; *** padj < |0.001|).

We compared the above-described brown bear muscle transcriptome to published transcriptomic data from a model of long-term physical inactivity in mice induced by 10 days of unloading, where the soleus muscle mass decreased by ~30% [42]. Comparing the two models, the muscle transcriptomic profiles appeared very different for selected gene expression related to TGF-β and BMP signaling pathways (Figure S1 and Tables S2–S4). Of note, gene expressions of TGF-β and BMP ligands were mainly differently regulated, as particularly evidenced for GDF5, which was up-regulated during bear hibernation, but down-regulated during unloading in the mouse model (FC = 0.18), and as well with MSTN strongly down-regulated during bear hibernation but unchanged in unloaded mouse muscles (FC = 1.57) (Figure 4a and Tables S2 and S3). Regarding receptor expressions, the two models responded quite similarly (Figure 4b and Tables S2 and S3), with three notable exceptions. Firstly, the gene expression of ACRV1C did not change in muscles of the unloaded mouse, unlike muscles of the hibernating bear, where a strong down-regulation occurred (FC = 1.11 and 0.28, respectively), what we also observed with the gene expression of TGFBR2 (FC = 1.08 and 0.80, respectively). Secondly, the gene expression of BMPR1B, GDF5 receptor, was up-regulated in muscles of the hibernating bear but was down-regulated in muscles of the unloaded mouse (FC = 0.66) (Figure 4b). The intracellular actors SMAD3, SMAD4, and SMAD1 were regulated similarly by bear hibernation or mouse unloading, although not to the same extent (FC = 0.73, 1.10, and 1.50, respectively). However, SMAD2 was up-regulated (FC = 1.24), and SMAD5 and SMAD9 remained unchanged only in the muscles of the unloaded mouse (Figure 4c).

**Figure 5:**
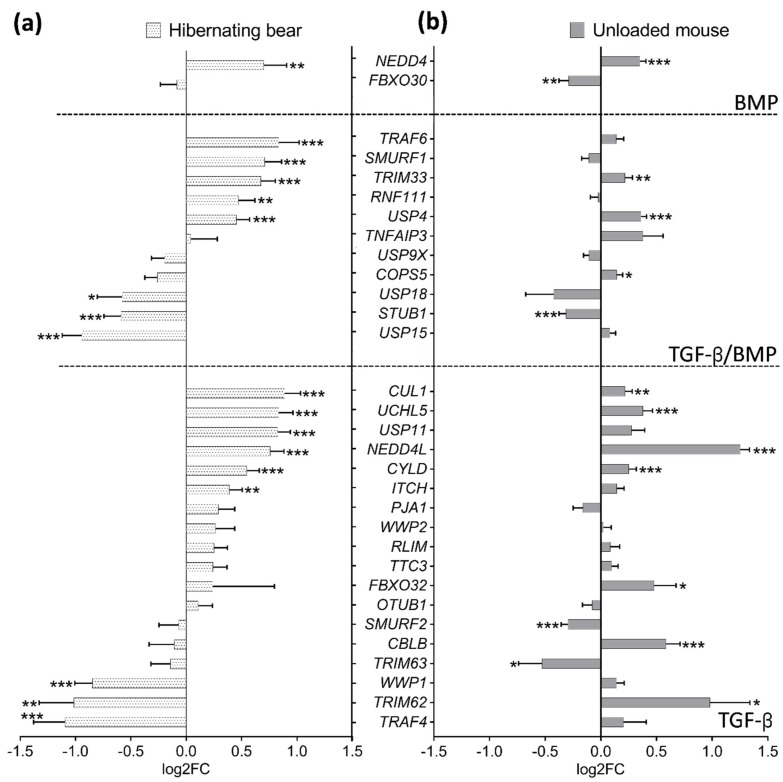
The gene expression pattern of muscle E3s/DUB enzymes involved in TGF-β and BMP pathway regulation differed in the atrophy-resistant brown bear muscle during hibernation compared to the atrophied muscle of the unloaded mouse. Genes expression level (**a**) in vastus lateralis muscle of active and hibernating brown bears (*n* = 6 bears/season, the same individuals were sampled and analyzed in summer and winter, log2FC Winter/Summer, dotted white bars), and (**b**) in soleus muscle of control and unloaded mice (*n* = 4 mice per condition, log2FC Unloaded/Control, gray bars). Data are expressed as log2FC ± lfcSE. Statistical significance is shown (* padj < |0.05|; ** padj < |0.01|; *** padj < |0.001|).

Regarding the E3s and DUB enzymes involved in the BMP pathway alone (Figure 5 upper panel), NEDD4 was similarly up-regulated in both models (FC = 1.63 and 1.28, respectively in bear and mouse), whereas FBXO30 (FC = 0.82) was down-regulated only in muscles of the unloaded mouse (Figure 5 and Table S4). The enzymes involved in the regulation of both TGF-β/BMP signaling were mainly up- or down-regulated in muscles of the hibernating bear but were mainly unaffected in unloaded mouse muscles (Figure 5 middle panel, Table S4). Finally, the E3s/DUB involved only in TGF-β signaling regulation were for half of them commonly unchanged or up-regulated, e.g., CUL1, UCHL5 or NEDD4L (FC = 1.17, 1.30 or 2.38, respectively), and for the other half oppositely regulated, e.g., SMURF2, CBLB, or TRIM62 (FC = 0.81, 1.50, and 1.97, respectively) in muscles of the unloaded mouse compared to muscles of the hibernating bear (Figure 5 lower panel and Table S4).

Overall, TGF-β and BMP signaling pathways were differentially regulated between atrophy-resistant muscles of the hibernating bear and atrophy-sensitive muscles of the unloaded mouse.

### 3.4. Hibernation Induces Changes in TGF-β and BMP Pathway Components at the Protein Level

**Figure 6:**
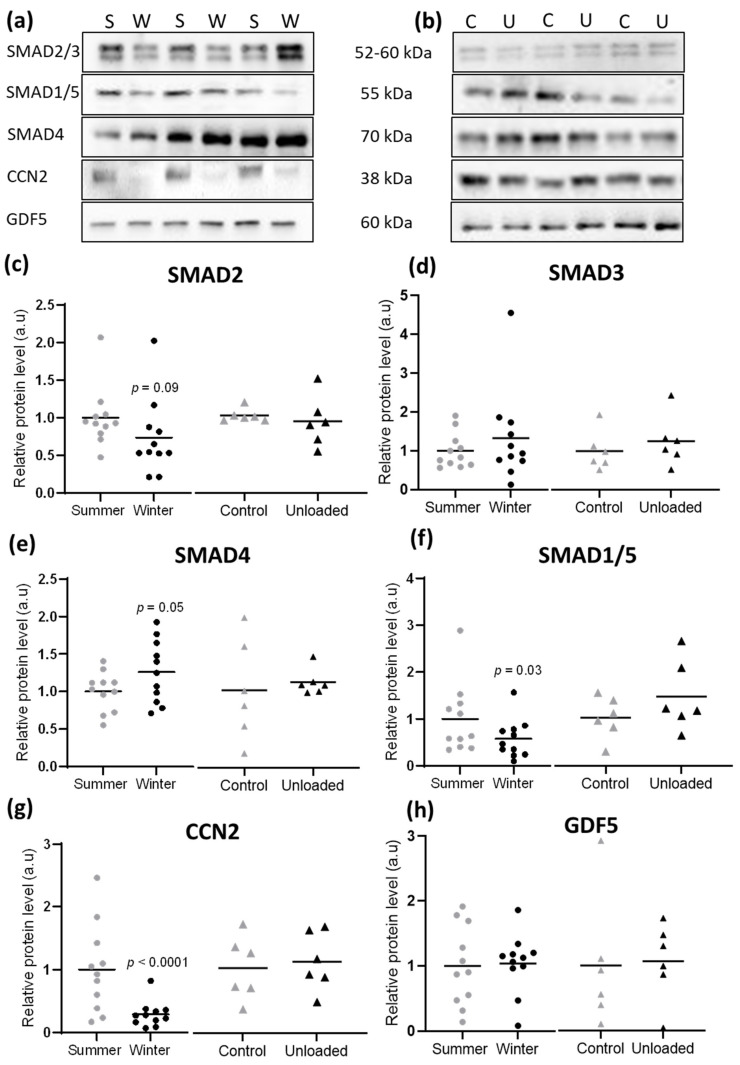
Hibernation induces changes in TGF-β and BMP components at protein level. Protein levels for total SMAD2/3, SMAD1/5, SMAD4, CCN2, and GDF5 were assessed by Western blots (**a**) in the vastus lateralis muscle of brown bears during summer (S) and winter (W) and (**b**) in the soleus muscle of control (C) and unloaded mice (U). Representative western blots are shown for three couples of bears and mice. (**c**–**h**). Data are presented as individuals’ values with mean bars (*n* = 11 bears/season, the same individuals were sampled and analyzed in summer and winter, and *n* = 6 mice per condition). Gray and black dots are for muscles of bears, in summer and winter, respectively, and gray and black triangles are for control and unloaded muscle of mice, respectively.

To further compare these models of resistance and vulnerability to atrophy, we explored the protein levels of the SMAD intracellular actors. In the brown bear muscle, we observed a tendency to decrease for SMAD2 protein levels (*p* = 0.09) in winter compared to summer, whereas SMAD3 levels remained quite similar between the two seasons (Figure 6a,c,d). Protein levels of SMAD4 were higher in muscles of the hibernating bear compared to the active one (Figure 6a,e), but the converse was observed for SMAD1/5 (Figure 6a,f). As for mRNA, these SMAD proteins did not follow the same regulation pattern in muscles of the unloaded mouse, where none changed at the protein level (Figure 6b–f). The protein levels of CCN2, a TGF-β target gene that is also an extracellular activator of the TGF-β pathway and an inhibitor of the BMP pathway, were strongly lower in winter bear muscles but were unaffected in unloaded mouse muscles (Figure 6a,b,g). Finally, the protein levels for GDF5, a BMP ligand, remained unchanged in both muscles from the hibernating bear and the unloaded mouse (Figure 6a,b,h).

## 4. Discussion

Although basic knowledge regarding the underlying mechanisms of muscle atrophy is continuously growing, essentially from rodent models and clinical studies in humans, there are still no efficient therapeutic strategies for its prevention and treatment. To explore new avenues, we compared a model of muscle atrophy resistance, the hibernating brown bear [23], and a mouse model of disuse-induced muscle atrophy [42]. Therefore, we analyzed the bear muscle transcriptome and identified sweeping changes in gene expression between the summer-active period and the winter-hibernating period, and we compared them with transcriptomic data from muscles of the unloaded mouse.

Whereas the loss of muscle mass during inactivity is often associated with a decrease in muscle protein synthesis [42,61,62,63], we reported here that genes implicated in protein metabolism were mainly up-regulated in muscles of the hibernating bear. This is consistent with previous studies that linked muscle atrophy resistance during hibernation to induction of protein translation [35,36,64]. Under activation of FOXOs transcription factors, atrogenes involved in both autophagy and UPS pathways are enhanced in rodents [65,66,67]. These atrogenes (e.g., *MAP1LC3A*, *FBXO32,* and *ZFAND5*) were indeed up-regulated or maintained in the unloaded mouse model but down-regulated or unchanged in muscles of the hibernating bear (Table S5). In agreement with previous studies, our data confirmed that proteolytic actors were up-regulated in disuse-induced muscle atrophy in rodents [11] but not in the bear model of muscle atrophy resistance [36]. Despite discrepancies between bear and mouse models (e.g., fed status, torpor vs. hindlimb muscle disuse), both display similar pathways controlling skeletal muscle mass and protein balance. For instance, the TGF-β signaling pathway has been reported to be evolutionarily conserved among several species, from *Caenorhabditis elegans* and *Drosophila melanogaster* to *Mus musculus* [68]. In addition, both models are responsive to denervation-induced atrophy when in active conditions [20,30]. We performed our analyses on the fast-twitch vastus lateralis muscle for the brown bear and the slow-twitch soleus muscle for the mouse. Thus, we cannot exclude the possibility that the differences recorded here between the models may partly have resulted from the specific nature of these muscles concerning their metabolic and contractile properties. However, it is noteworthy that, although fast-twitch muscles are not as sensitive to physical inactivity as slow-twitch muscles, both types of muscles atrophied in classical models of long-term physical inactivity in rodents [25,29,39,69,70] and humans [71,72,73]. Despite this difference in metabolic and contractile properties, a general down-regulation of genes involved in extracellular matrix (ECM) structure organization was observed in atrophied muscles from the unloaded mouse and atrophy-resistant muscles from the hibernating brown bear (Figure 1 and Table S2). This ECM structure remodeling is a common feature of other atrophic models reported during dystrophic diseases [74] or muscle disuse in rodents or humans [26,69,75,76,77].

TGF-β is currently of major interest within the field of skeletal muscle biology. Indeed, the gene disruption of two of its ligands Myostatin (*MSTN*) or Activin A (*INHBA*), and the inhibition of their shared receptor ActRIIB (*ACVR2B*) promote a profound muscle hypertrophy phenotype in various conditions and species [78,79,80,81]. On the contrary, over-expression of *MSTN* or *INHBA* leads to the recruitment and phosphorylation of SMAD2-3, triggering an atrophic transcriptional program [16,17,82,83,84]. Here, we reported that the expression levels of both ligands *MSTN* and *INHBA* were lower only in muscles of the hibernating bear. In addition, *ACVR2B* and *SMAD2* mRNA levels were up-regulated in muscles of the unloaded mouse whereas maintained in muscles of the hibernating bear. At the protein level, SMAD2 was maintained in the muscles of the unloaded mouse and showed a tendency to decrease in muscles of the hibernating bear. However, despite extensive efforts and numerous antibodies tested, we could not characterize the SMADs phosphorylation status in bear muscles, which thus remains to be defined.

One TGF-β target gene, CCN2 (also known as CTGF), is an ECM protein associated with fibrotic activity that is up-regulated in several muscle chronic disorders (i.e., Duchenne muscular dystrophy or the amyotrophic lateral sclerosis) [85]. CCN2 is one of the main pro-fibrotic cytokines acting downstream of TGF-β signaling and can amplify its effects through enhancement of TGF-β ligand-receptor binding [85,86,87,88,89,90]. Inhibition of *CCN2* gene expression reduced fibrosis and improved muscle and locomotor performance in a rodent model of amyotrophic lateral sclerosis [86]. During unloading, mRNA and protein levels of CCN2 were unchanged in the muscles of the unloaded mouse but were strongly lower in the muscles of the hibernating brown bear. Taken together, our data strongly suggest that TGF-β signaling is overall inhibited only in muscles resistant to atrophy during bear hibernation.

The BMP pathway is a potent inducer of bone and cartilage formation [91]. BMP signaling was also recently discovered as a regulator of muscle mass, as its inhibition abolished the hypertrophic phenotype of the *MSTN*-KO mouse [20], and an increase in its receptor activity induced important muscle hypertrophy [21]. Regarding BMP ligands, GDF5 is essential to muscle mass maintenance, binding preferentially to the type I receptor BMPR1B. GDF5 expression was strongly induced in denervated mouse muscles, and its inhibition worsened muscle atrophy, suggesting a role in counteracting denervation-induced atrophy [20]. We report here that the gene expression for both *GDF5* and *BMPR1B* was strongly down-regulated in mouse muscles during unloading, whereas up-regulated in muscles of the hibernating bear. However, GDF5 protein levels were stable in the muscles of both the unloaded mouse and the hibernating bear. We recently demonstrated that circulating components of the hibernating bear serum were able to induce trans-species effects on human myotubes, notably inhibition of protein degradation. Therefore, we hypothesized that those components could be involved in the maintenance of muscle mass and strength in the hibernating bear [92]. Along with muscle mass maintenance, bear hibernation is also associated with bone mass maintenance [93], and thus GDF5 may constitute a possible target toward muscle and bone protection during long periods of physical inactivity and/or fasting. Unfortunately, the exploration of GDF5 concentration in bear serum was hampered by species cross-reactivity concerns with commercially available Elisa kits and thus remains to be addressed.

It has been proposed that the BMP and TGF-β common actor SMAD4 mainly engages with the TGF-β pathway, but switches to the BMP pathway when TGF-β transduction is reduced, and thus could be the limiting factor between these two signalings [20]. Moreover, denervation-induced muscle atrophy was exacerbated in the SMAD4 deficient mouse [20], and muscle mass was increased in humans with a mutation-associated gain of function in the SMAD4 gene [94]. We observed here higher mRNA and protein levels for SMAD4 in muscles of the hibernating bear, but only at mRNA levels in muscles of the unloaded mouse. This is concomitant with the overall down-regulation highlighted for the TGF-β signaling in the hibernating bear, which was not observed for the unloaded mouse. Thus, the inhibition of the TGF-β signaling in muscles of the hibernating bear may have released SMAD4 from TGF-β to BMP pathway to maintain muscle mass in a long period of disuse (Graphical abstract).

TGF-β and BMP share a SMAD-independent pathway that activates the E3 ubiquitin ligase TRAF6 [58]. In addition to its pro-atrophic role [95], TRAF6 is also required for myogenic differentiation and muscle regeneration via the MEF2 axis [96]. MEF2 is a conserved family of transcription factors involved in the control of muscle gene expression [97]. A recent muscle transcriptome analysis highlighted an inhibition of MEF2 transcription factors during human bed rest leading to skeletal muscle alterations [98]. Here, only *MEF2A* was up-regulated during unloading in mouse muscles, whereas *TRAF6*, *MEF2A,* and *MEF2C* were up-regulated in the hibernating bear muscles. We already observed a myogenic microRNA signature mediated by MEF2A signaling in the muscles of the hibernating bear, promoting mechanisms of muscle regeneration, suppression of ubiquitin ligases, and resistance to muscle atrophy [33]. Further studies are required to address whether this MEF2 signature could be under the control of the TGF-β and/or the BMP pathway through TRAF6.

## 5. Conclusions

Resistance to muscle atrophy in hibernating brown bears has so far been linked to a reduction in protein and energy metabolism. Here, we show for the first time that the TGF-β pathway is down-regulated whereas the BMP pathway is concomitantly sustained or even up-regulated in atrophy-resistant muscles of the hibernating brown bear. Thus, beyond strengthening the previous hypothesis of a hypometabolism enabling this natural resistance to muscle atrophy, our study provides new insights regarding the underlying mechanisms.

The originality of the current work lies in the choice to study the mechanisms involved in resistance to atrophy, and not solely, as in many studies, the mechanisms involved in the onset of atrophy. Our comparison of resistance and sensitivity to muscle atrophy animal models suggested the balance between the TGF-β and the BMP pathways as critical for preventing skeletal muscle atrophy over a long period of disuse. Many targeted therapies to counteract muscle atrophy already focus on TGF-β inhibition [99]. Our data open the way for further studies and clinical trials to test the effects of strategies to switch on (or sustain) the BMP pathway in combination with TGF-β inhibition to prevent disuse-induced muscle atrophy.

## Data Availability

The analyzed transcriptomic mouse data that support the findings of this study are openly available in the GEO repository database at https://doi.org/10.1093/gerona/gly051, accessed on 16 June 2021, reference number (GSE102284). The generated transcriptomic bear data that support the findings of this study are openly available in the GEO repository database at https://www.ncbi.nlm.nih.gov/geo/query/acc.cgi, accessed on 16 June 2021, reference number (GSE144856). Other data that support the findings of this study are available in the supplementary material of this article.

## Supplementary Materials

The following are available online at https://www.mdpi.com/article/10.3390/cells10081873/s1, Figure S1, Tables S1–S6, and Blots quantification.
