# Supplementary material for: Concurrent BMP Signaling Maintenance and TGF-β Signaling Inhibition Is a Hallmark of Natural Resistance to Muscle Atrophy in the Hibernating Bear
Source: Cells. 2021 Jul 23;10(8):1873. doi: 10.3390/cells10081873 (PMC8393865; doi:10.3390/cells10081873)
Supplement: Supplementary file 1 [file cells-10-01873-s001.zip › cells-1282632-supplementary/Supplementary Figure 1.pdf]

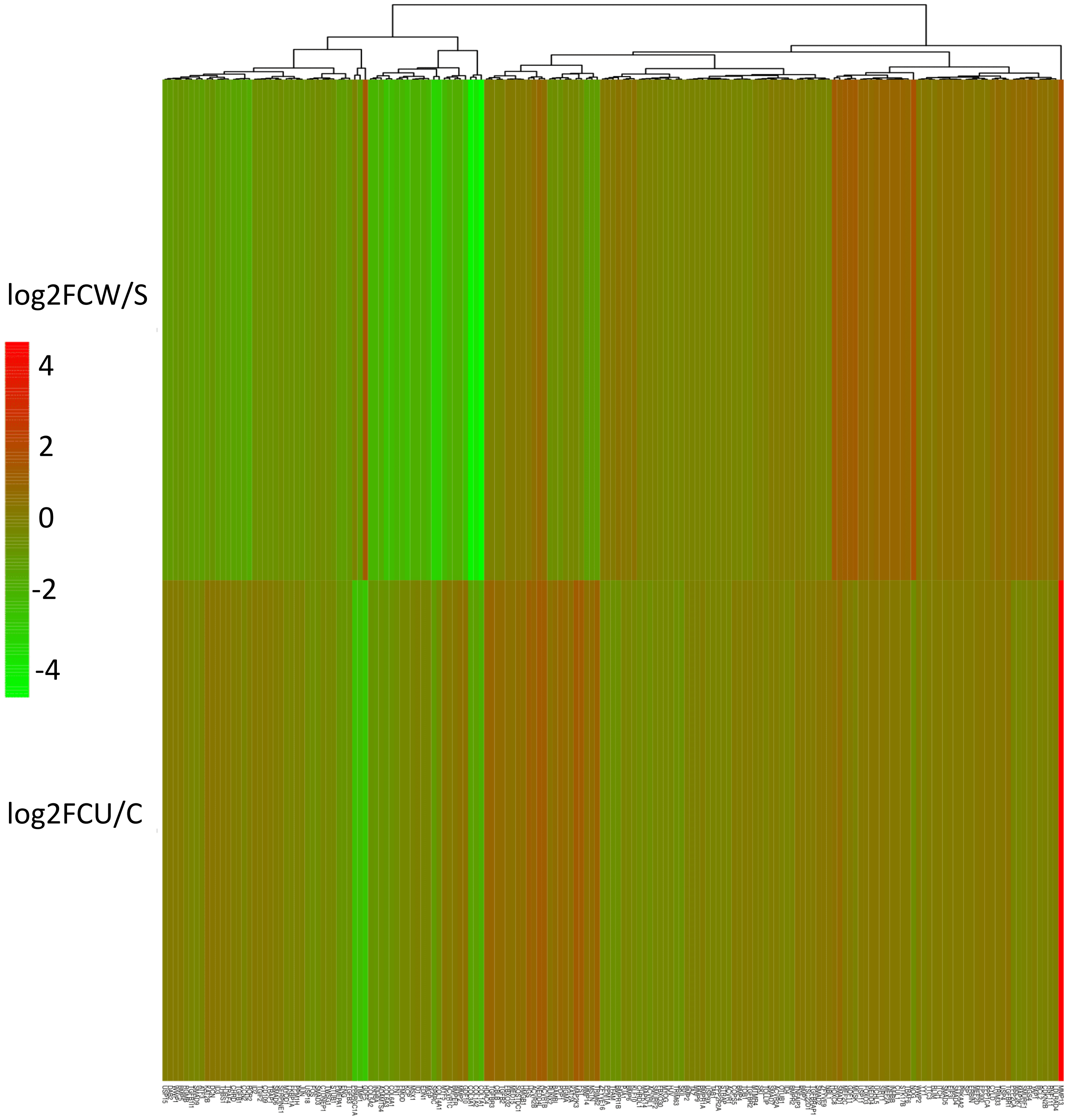

Supplementary Figure S1

Supplementary Figure 1. The gene expression pattern of TGF-β and BMP components is different in brown bear muscle resistant to atrophy during hibernation compared to atrophied muscles of the unloaded mouse. Heatmap from vastus lateralis muscle of active and hibernating brown bears (n=6 bears/season, the same individuals were sampled and analyzed in summer and winter, log2FCWinter/Summer), and soleus muscle of control and unloaded mice (n=4 mice per condition, log2FC Unloaded/Control) of 171 TGF-β/BMP related genes. The green and red colours indicate that the gene expression decreased or increased, respectively, and each line represents one gene.
